# Impact of cryoablation on operative outcomes in thoracotomy patients

**DOI:** 10.1093/icvts/ivae023

**Published:** 2024-02-10

**Authors:** Kian Pourak, Rachel Kubiak, Kumaran Arivoli, Kiran Lagisetty, William Lynch, Jules Lin, Andrew Chang, Rishindra M Reddy

**Affiliations:** University of Michigan Medical School, Ann Arbor, MI, USA; University of Michigan Medical School, Ann Arbor, MI, USA; University of Michigan Medical School, Ann Arbor, MI, USA; University of Michigan Medical School, Ann Arbor, MI, USA; Section of Thoracic Surgery, Department of Surgery, Michigan Medicine, Ann Arbor, MI, USA; University of Michigan Medical School, Ann Arbor, MI, USA; Section of Thoracic Surgery, Department of Surgery, Michigan Medicine, Ann Arbor, MI, USA; University of Michigan Medical School, Ann Arbor, MI, USA; Section of Thoracic Surgery, Department of Surgery, Michigan Medicine, Ann Arbor, MI, USA; University of Michigan Medical School, Ann Arbor, MI, USA; Section of Thoracic Surgery, Department of Surgery, Michigan Medicine, Ann Arbor, MI, USA; University of Michigan Medical School, Ann Arbor, MI, USA; Section of Thoracic Surgery, Department of Surgery, Michigan Medicine, Ann Arbor, MI, USA

**Keywords:** Thoracotomy, Pain management, Cryoablation, Epidural, Opioid use

## Abstract

**OBJECTIVES:**

Cryoablation is increasingly being utilized as an alternative to epidurals for patients undergoing thoracotomies. Current evidence suggests cryoablation may decrease postoperative analgesia utilization, but could increase operative times. We hypothesized that the adoption of intraoperative cryoablation to manage post-thoracotomy pain would result in reduced length of stay and reduced perioperative analgesia compared to routine epidural use.

**METHODS:**

A retrospective analysis was performed from a single, quaternary referral centre, prospective database on patients receiving thoracotomies between January 2020 and March 2022. Patients undergoing transthoracic hiatal hernia repair, lung resection or double-lung transplant were divided between epidural and cryoablation cohorts. Primary outcomes were length of stay, intraoperative procedure time, crossover pain management and oral narcotic usage the day before discharge.

**RESULTS:**

During the study period, 186 patients underwent a transthoracic hiatal hernia repair, lung resection or double-lung transplant with 94 receiving a preoperative epidural and 92 undergoing cryoablation. Subgroup analysis demonstrated no significant differences in demographics, operative length, length of stay or perioperative narcotic use. Notably, over a third of patients in each cryoablation subgroup received a postoperative epidural (45.5% transthoracic hiatal hernia repair, 38.5% lung resection and 45.0% double-lung transplant) for further pain management during their admission.

**CONCLUSIONS:**

Cryoablation use was not associated with an increase in procedure time, a decrease in narcotic use or length of stay. Surprisingly, many cryoablation patients received epidurals in the postoperative period for further pain control. Additional analysis is needed to fully understand the benefits and costs of epidural versus cryoablation strategies.

**Figure:**
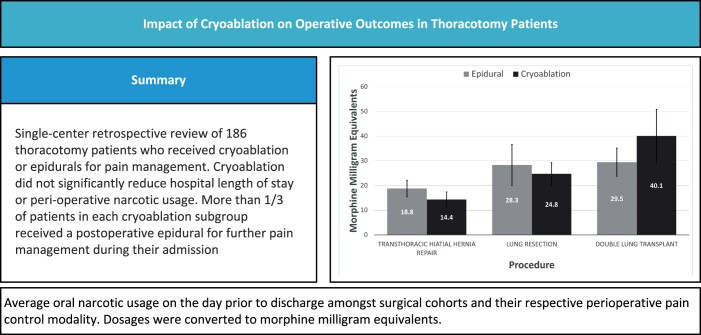

## INTRODUCTION

Posterolateral thoracotomy incisions have been the standard approach for thoracic surgeons due to their excellent exposure of the chest [1]. However, this method of intraoperative exposure requires significant rib retraction and possible rib resection, which can result in increased postoperative pain secondary to damage to intercostal nerves [2]. Thoracotomy incisions have been associated with higher rates of narcotic use compared to other surgical incisions [3], and there are multiple efforts being undertaken to reduce postoperative narcotic use [4, 5]. Furthermore, post-thoracotomy pain syndrome can result in chronic pain issues for many patients and increase the risk of prolonged use of narcotic pain medications [6, 7].

A variety of pain management techniques have been utilized to minimize postoperative pain from thoracotomies, but it is not fully understood which methods optimize outcomes in the acute and chronic settings for patients. Epidurals are a commonly utilized method of analgesia that act at nerve roots to limit the stress response associated with surgical procedures [8]. By initiating and maintaining analgesic action prior to and after the onset of stimuli from surgery, preoperative epidural placement has been shown to reduce acute post-thoracotomy pain and complications such as pneumonia [9, 10]. Our institutional practice for over a decade has relied on preoperative epidural placements for elective thoracotomies. However, epidural usage has a risk of adverse outcomes, such as haematoma, infection and postoperative hypotension, which can negatively impact patient care [11, 12]. In our experience, we have struggled with operative delays, perioperative epidural function, postoperative hypotension and postoperative urinary retention secondary to epidural placement.

More recent studies have shown that multimodal therapies, as part of ‘enhanced recovery after surgery’ protocols, can also reduce acute pain [13–15]. These focus on addressing pain through a combination of medications to reduce inflammation, nerve-related pain signals and postoperative stress response [16]. Another alternative method for acute pain management after thoracotomy is intercostal cryoanalgesia, which involves intraoperative freezing of intercostal nerves. The process involves placing a probe directly in the intercostal space and utilizing cold temperatures to induce cell damage and disrupt nerve conduction [17]. The benefits of this technique are that it does not result in permanent nerve damage and is associated with improved pain outcomes and reduced opioid use in both thoracotomy and thoracic surgery patients [18–20]. We hypothesized that the adoption of a cryoablation process, as an alternative to preoperative epidural placement, to treat post-thoracotomy pain would result in reduced length of stay and reduced perioperative narcotic use, compared to patients receiving epidurals.

## MATERIALS AND METHODS

### Ethics statement

This study was Institutional Review Board (IRB) approved and the data collection fell between 2 different database protocols (HUM00047546, approved March 2011, and HUM00042792, approved January 2011). These are ongoing research IRBs allowing for the analysis of outcomes after thoracic surgery.

### Patient selection

A retrospective review was performed from a single, quaternary referral centre prospective database on patients receiving thoracotomies between January 2020 and March 2022. Patients were included if they underwent a transthoracic hiatal hernia repair (TTHHR), lung resection (LR) or double-lung transplant (DLT) and were alive upon discharge. Exclusion criteria included patients who had a history of substance use disorder before admission or died during admission. Cohorts were subdivided between patients who received a preoperative epidural versus intraoperative cryoablation. Perioperative pain control management was decided on by the surgeon. Six surgeons performed the operations, with 3 of them being adopters of the cryoablation approach. For patients who received preoperative epidurals, the epidurals were placed by anaesthesia providers in the preoperative holding area for elective procedures, and within 24–48 h of surgery for lung transplants. The medications used were a combination of bupivacaine with an opioid, either hydromorphone or fentanyl. There were standard baseline levels of medication delivered with a patient-controlled additional dose allowed every 12–15 min. The routine cryoablation was performed by the thoracic surgeon before the primary closure of thoracotomies. All patients were part of enhanced recovery after surgery pathways, which included preoperative ambulation, early ambulation after surgery, incentive spirometry and patient-controlled analgesia.

### Operative technique

For patients receiving cryoablation, a routine posterolateral thoracotomy was performed in TTHHRs and LRs, while a bilateral anterior thoracotomy was performed in DLTs. Prior to closure, 7–10 ml of 0.5% Marcaine was injected subpleurally for 5 intercostal spaces with a 25 gauge needle, 2 spaces above the thoracotomy, the thoracotomy space and 2 spaces below. The local anaesthetic was utilized to provide acute pain management prior to the onset of cryoablation’s pain management, which requires approximately 24–48 h to take effect. Following this, the Cryosphere S probe (Atricure ©) was used to administer 120 s of cryotherapy to each space, with the temperature reaching –70°C. The probe, which has an 8 mm diameter, was placed approximately 3 cm away from the vertebral body and in the middle of the intercostal space. This paradigm is in line with the manufacturer’s instructions. Once all 5 intercostal spaces were treated, the closure was resumed in a normal fashion. The overall process was estimated to take 15 min intraoperatively per side. The use of the local anaesthetic was begun after the 1st few months of adopting the cryoablation due to recognition of poorer pain control in the first day after surgery.

### Data collection

Pre-, intra- and postoperative data elements were obtained from the Society of Thoracic Surgery from Michigan Medicine’s Cardiac Surgery Warehouse. Data collection was supplemented with a retrospective review of the patients’ medical records and operative reports. Primary outcomes were defined as the length of hospital stay, intraoperative procedure time, crossover pain management and oral narcotic usage the day before discharge. Intraoperative procedure time was defined as the time between the initial skin incision and completion of the wound dressing. Crossover pain management was pertinent for the cryoablation cohorts. Charts were reviewed to determine if patients who had not previously received an epidural in the preoperative period had one placed postoperatively for multimodal pain management. Oral narcotic usage the day before discharge was obtained through record review and calculated as total measured morphine milligram equivalents (MME) per day [21].

### Statistical analysis

Univariate comparisons between epidural and cryoablation cohorts were performed using Fisher’s exact tests for categorical variables and Wilcoxon rank sum tests for continuous variables. Bonferroni corrections were completed for comparison of epidural versus cryoablation in the TTHHR, LR and DLT subgroups. The normality of data in each cohort was assessed using Shapiro–Wilk tests, which revealed a non-normal distribution for the data sets. Data analysis was performed using MATLAB (Mathworks, Natick, MA). Data were presented as mean (standard deviation) for continuous data and *n* (%) for categorical variables.

## RESULTS

### Cohort and demographic data

During the study period, 297 patients received thoracotomies. Of these, 186 patients underwent a TTHHR, LR or DLT. Subgroups included 76 TTHHR [43 epidural (E) vs 33 cryoablation (C)], 58 LR (19 E vs 39 C) and 52 DLT (32 E vs 20 C).

The patients in each subgroup had similar ages and no significant differences in gender. Pre-existing comorbidities, including hypertension, coronary artery disease, cerebrovascular disease, diabetes and liver disease, amongst subgroups were similar. There was a significantly higher number of non-smokers amongst the TTHHR epidural cohort versus TTHHR cryoablation cohort (83.7 vs 57.6%, *P* < 0.05). However, there were no significant differences in former smokers, current smokers or alcohol use disorder within other subgroups (Table 1).

**Table 1:** Descriptive statistics amongst cryoablation versus epidural pain management cohorts

	Total	Transthoracic hiatal hernia repair			Lung resection			Double-lung transplant		
		Epidural	Cryoablation	*P*-value	Epidural	Cryoablation	*P*-value	Epidural	Cryoablation	*P*-value
*n*	186	43 (56.6)	33 (43.4)		19 (32.8)	39 (67.2)		32 (61.5)	20 (38.5)	
Age, mean years ± SD	61.5 ± 11.4	66.5 ± 9.8	64.1 ± 11.6	0.34	59.7 ± 12.3	63.4 ± 10.8	0.25	54.8 ± 9.2	55.1 ± 8.7	0.91
Gender, *n* (%)										
Male	83 (44.6)	10 (23.3)	7 (21.2)	0.53	12 (63.2)	23 (59.0)	0.49	17 (53.1)	14 (70.0)	0.93
Female	103 (55.4)	33(76.7)	26 (78.8)	0.69	7 (36.8)	16 (41.0)	0.72	15 (46.9)	6 (30.0)	0.18
Race, *n* (%)										
White/Caucasian	171 (91.9)	40 (93.0)	33 (100)	>0.99	19 (100)	35 (89.7)	0.19	26 (81.3)	18 (90.0)	0.90
Black/African American	8 (4.3)	1 (2.3)	0 (0)	0.57	0 (0)	2 (5.1)	>0.99	4 (12.5)	1 (5.0)	0.35
Other	7 (3.8)	2 (4.7)	0 (0)	0.33	0 (0)	2 (5.1)	>0.99	2 (6.3)	1 (5.0)	0.67
Pre-existing comorbidities, *n* (%)										
Hypertension	89 (47.8)	24 (55.8)	18 (54.5)	0.55	9 (47.4)	21 (53.8)	0.77	10 (31.3)	7 (35.0)	0.72
CAD	20 (10.8)	3 (7.0)	2 (6.1)	0.63	1 (5.3)	6 (15.4)	0.95	6 (18.8)	2 (10.0)	0.33
CVD	19 (10.2)	4 (9.3)	5 (15.2)	0.57	2 (10.5)	5 (12.8)	0.74	0 (0)	3 (15.0)	>0.99
Diabetes	27 (14.5	6 (14.0)	3 (9.1)	0.39	3 (15.8)	8 (20.5)	0.78	4 (12.5)	3 (15.0)	0.75
Liver disease	8 (4.3)	1 (2.3)	1 (3.0)	0.76	1 (5.3)	2 (5.1)	0.70	3 (9.4)	0 (0)	0.22
Substance use history, *n* (%)										
Non-smoker	83 (44.6)	36 (83.7)	19 (57.6)	0.04	0 (0)	9 (23.1)	0.06	12 (37.5)	7 (35.0)	0.55
Former smoker	94 (50.5)	7 (16.3)	13 (39.4)	0.30	16 (84.2)	25 (64.1)	>0.99	20 (62.5)	13 (65.0)	0.68
Current smoker	8 (4.3)	0 (0)	1 (3.0)	>0.99	3 (15.8)	4 (10.3)	0.41	0 (0)	0 (0)	>0.99
Alcohol use disorder	2 (1.1)	0 (0)	0 (0)	>0.99	2 (10.5)	0 (0)	0.10	0 (0)	0 (0)	>0.99

CAD: coronary artery disease; CVD: cerebrovascular disease; SD: standard deviation.

### Operative and perioperative outcomes

**Figure 1:**
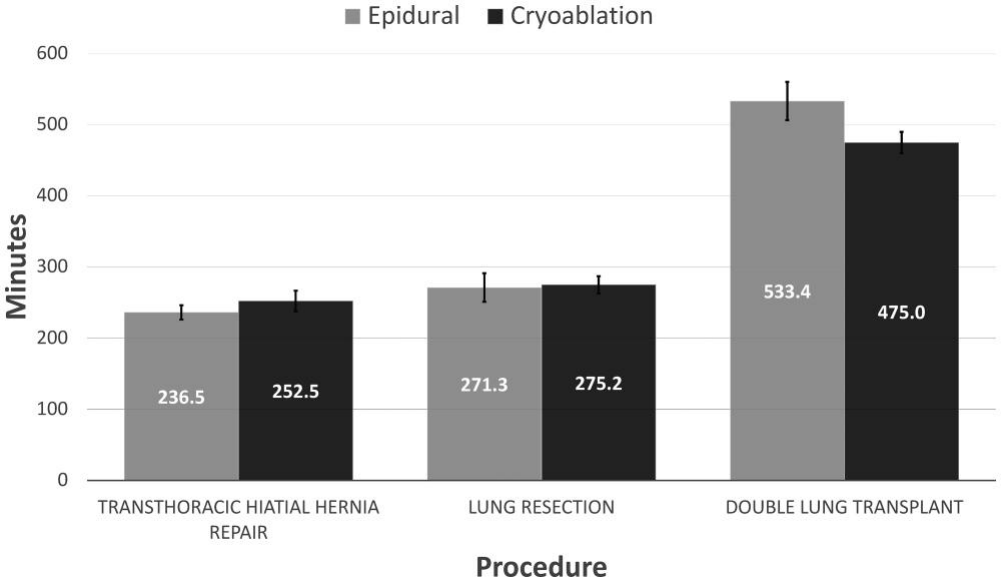
Average operation length amongst surgical cohorts and their respective perioperative pain control modalities.

Operative outcomes were similar between the epidural and cryoablation cohorts within each subgroup. The use of cryoablation was not associated with a statistically significant increase in procedure time amongst the TTHHR [252.5 (SD 83.5) C vs 236.5 (SD 66.7) E min, *P* = 0.56], LR [275.2 (SD 74.7) C vs 271.3 (SD 87.1) E min, *P* = 0.82] and DLT [475.0 (SD 67.3) C vs 533.4 (SD 152.0) E min, *P* = 0.14] when compared to the epidural cohort (Fig. 1).

**Figure 2:**
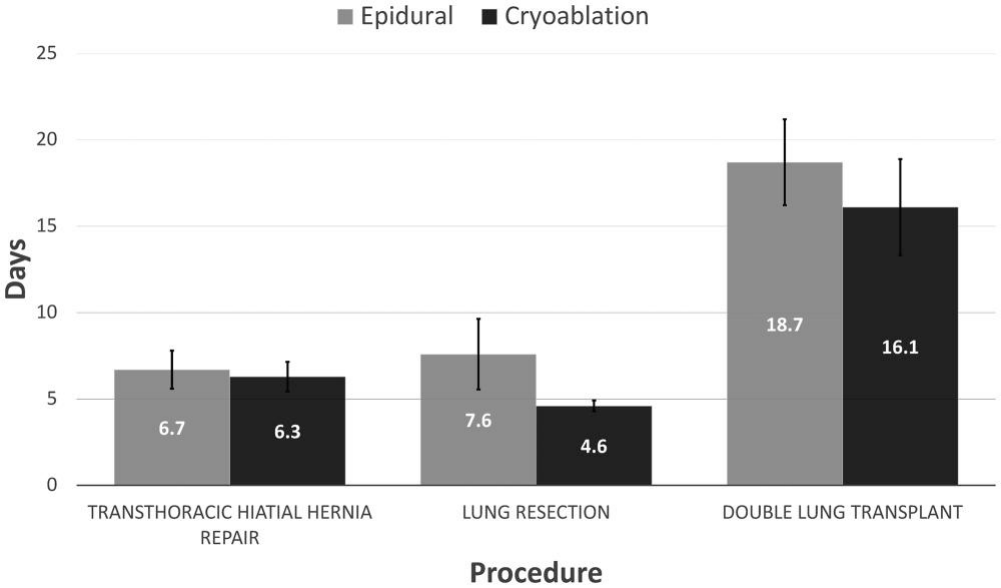
Average duration between operation and discharge amongst surgical cohorts and their respective perioperative pain control modalities.

**Figure 3:**
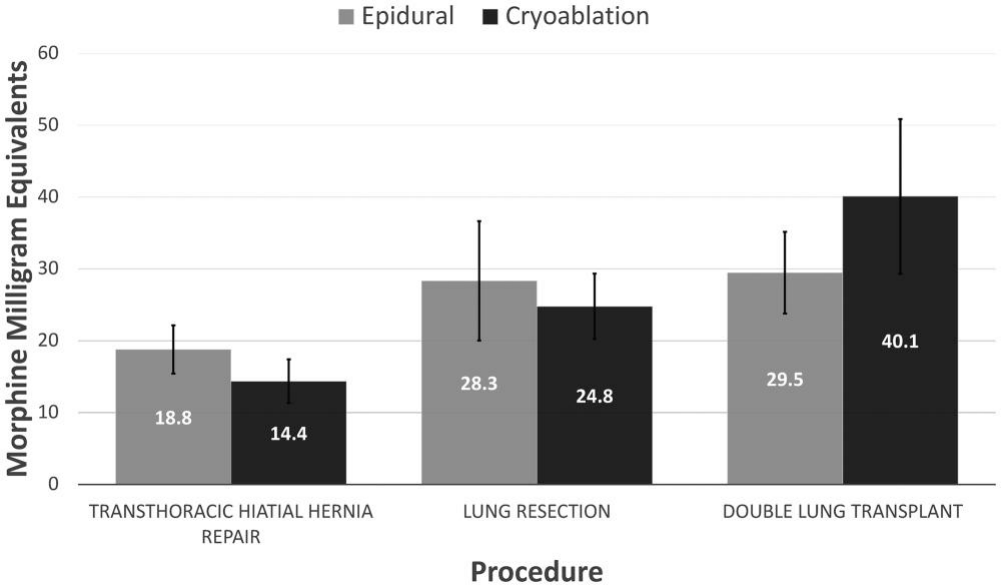
Average oral narcotic usage on the day prior to discharge amongst surgical cohorts and their respective perioperative pain control modality. Dosages were converted to morphine milligram equivalents.

Additionally, the perioperative outcomes amongst the epidural and cryoablation subgroups demonstrated no significant differences (Table 2). Cryoablation was not associated with a reduced length of stay for patients who underwent TTHHR [6.3 (SD 4.9) C vs 6.7 (SD 7.2) E days, *P* = 0.43], LR [4.6 (SD 1.9) C vs 7.6 (SD 8.9) E days, *P* = 0.30] or DLT [16.1 (SD 12.4) C vs 18.7 (SD 14.1) E days, *P* = 0.38] (Fig. 2). Additionally, oral narcotic usage the day before discharge was not lower amongst cryoablation patients in the TTHHR [14.4 (SD 17.5) C vs 18.8 (SD 22.0) E MME, *P* = 0.48], LR [24.8 (SD 30.1) C vs 28.3 (SD 36.2) E MME, *P* = 0.96] and DLT [40.1 (SD 48.2) C vs 29.5 (SD 32.1) E MME, *P* = 0.81] subgroups (Fig. 3). Furthermore, cryoablation did not decrease the number of patients in the TTHHR (84.8% C vs 95.3% E, *P* = 0.12), LR (89.7% C vs 89.5% E, *P* = 0.70) or DLT (80.0% C vs 90.6% E, *P* = 0.25) subgroups that were discharged with oral narcotics. Notably, over a third of patients in each cryoablation subgroup received a postoperative epidural (45.5% TTHHR, 38.5% LR and 45.0% DLT) for further pain management during their admission. There was no difference in rates of postoperative epidural placement over the time period of the study. Further analysis showed similar proportions of cryoablation patients received crossover pain management between 2020 and 2021–2022 in the TTHHR (41.6 vs 55.6%, *P* = 0.79), LR (55.6 vs 34.5%, *P* = 0.23) and DLT (33.3 vs 54.5%, *P* = 0.42) cohorts.

**Table 2:** Primary outcomes amongst cryoablation versus epidural pain management cohorts

	Transthoracic hiatal hernia repair			Lung resection			Double-lung transplant		
	Epidural	Cryoablation	*P*-value	Epidural	Cryoablation	*P*-value	Epidural	Cryoablation	*P*-value
*n* (%)	43 (56.6)	33 (43.4)		19 (32.8)	39 (67.2)		32 (61.5)	20 (38.5)	
Procedure duration (min), mean (SD)	236.5 (66.7)	252.5 (83.5)	0.56	271.3 (87.1)	275.2 (74.7)	0.82	533.4 ( 152.0)	475.0 (67.3)	0.14
Length of admission (days), mean (SD)	6.7 (7.2)	6.3 (4.9)	0.43	7.6 (8.9)	4.6 (1.9)	0.30	18.7 (14.1)	16.1 (12.4)	0.38
Oral narcotic usage day before discharge (morphine milligram equivalents), mean (SD)	18.8 (22.0)	14.4 (17.5)	0.48	28.3 (36.2)	24.8 (30.1)	0.96	29.5 (32.1)	40.1 (48.2)	0.81
Discharged with narcotics, *n* (%)	41 (95.3)	28 (84.8)	0.12	17 (89.5)	35 (89.7)	0.70	29 (90.6)	16 (80.0)	0.25
Additional epidural placed after surgery, *n* (%)		15 (45.5)			15 (38.5)			9 (45.0)	

SD: standard deviation.

## DISCUSSION

In this retrospective, non-randomized case–cohort review, we compared outcomes in patients undergoing cryoablation versus epidurals for thoracotomies. We found no significant association between cryoablation and a decreased postoperative hospital length of stay. Additionally, there were no associations between cryoablation and increased procedure times or decreased narcotic use prior to discharge when comparing specific operations.

Interestingly, our data showed that significant portions of patients (45.5% TTHHR, 38.5% LR and 45.0% DLT) receiving cryoanalgesia also received postoperative epidural placement to further address pain concerns. Early in our experience, we were not routinely giving intrapleural local anaesthesia prior to the cryoablation. We sought to analyse if there was a difference in post-ablation epidural placement based on the use of local anaesthetic, but did not see a temporal difference in the reduced need for epidural placement after cryoablation. One issue of note is that we did not change our other postoperative pathways during this study period (use of toradol, intravenous Tylenol or other adjuncts). We have since employed a modified recovery after surgery pathway with anecdotally less postoperative epidural placement. Axtell *et al.* [22] compared postoperative pain outcomes in thoracic surgery patients receiving epidurals in the preoperative versus postoperative periods. Their results showed reduced patient-reported pain scores in the first 72 h after surgery for the preoperative epidural group. This demonstrates that preoperative epidural placement may alleviate more immediate postoperative pain compared to the delayed placement of a postoperative epidural after initial cryoablation failed to sufficiently manage pain. Additionally, evidence shows that the aggressive management of early postoperative pain helps reduce chronic pain in thoracotomy patients [23]. We hope that our modified pathways will help with postoperative pain control.

Different studies have evaluated the utility of cryoablation in other thoracic procedures. A recent meta-analysis from Daemen *et al.* analysed cryoablation efficacy in the Nuss procedure used to treat pectus excavatum. Among the 5 studies included, it was shown that cryoablation is associated with shortened postoperative length of stay [24]. Additionally, qualitative analysis showed decreased postoperative opioid usage with cryoablation in a majority of the studies. In contrast, Clemence *et al.* evaluated cryoablation in open thoracic aortic aneurysm and thoraco-abdominal aortic aneurysm repair. Here, results demonstrated that narcotic usage was significantly reduced in the cryoablation group; however, cryoablation was associated with increased postoperative length of stay and a longer time for extubation [25].

Our study did not reveal an increase in operative times with cryoablation. We had hypothesized that the addition of 10–15 min per side would result in consistently longer operative times, but we did not find statistically significant longer operative times. These data are inconsistent with prior studies that have shown longer operative times associated with cryoablation use [26, 27]. However, we also noted that cryoablation could be performed concurrently while other procedures, such as chest tube placement or rib fixation, were being completed, which may explain the discrepancy between our results as compared to prior studies. We also had different surgeons who preferred the cryoablation versus epidurals, which could contribute to this lack of difference.

Low sample sizing also may help explain some of the discrepancies among the previous studies. Among all studies evaluated in the Daemen *et al.* meta-analysis, none had more than 35 total participants. In the paper by Clemence *et al.*, there were only 25 total patients who received cryoablation. Another potential explanation for the discrepancies in cryoablative efficacy among various thoracic procedures may be from the relative invasiveness of the procedures themselves. The Nuss procedure mentioned above is a minimally invasive procedure, with prior literature supporting the use of cryoablation over epidural placement. In contrast, the procedures mentioned in the Clemence *et al.*’s study, in addition to those in our present study, pertain to more invasive thoracotomies, where cryoablation is not associated with better pain outcomes. This is further supported by a recent meta-analysis in which thoracic epidural usage was generally preferred over cryoablation among the 4 studies that investigated pain outcomes in thoracotomies [28]. We thus hypothesize that the efficacy of cryoablation in thoracic surgery procedures may exist on a spectrum, whereby its utility in managing pain increases with less-invasive operations or only select patients may benefit from the cryoablation procedure. Further work is needed to validate and explain this hypothesized relationship.

Our study has a number of limitations including small sample size in the subgroups, which can contribute to low statistical power. The use of the cryoablation was surgeon-dependent, which contributed to a selection bias. We are working on creating a randomized control protocol to evaluate the cryoablation protocol prospectively. Another limitation includes the lack of standardization of intrapleural local anaesthesia administration prior to cryoablation during the start of the study or the adoption of other recovery adjuncts like toradol or IV Tylenol, which we are using now, but beyond the time period of this study. Additionally, the study’s measurement of perioperative narcotic usage only captures a snapshot of patients’ postoperative pain. Finally, our study did not include any patient-reported data, which can provide valuable insight into their experiences of postoperative outcomes.

## CONCLUSION

Our works suggests that the use of cryoablation during thoracotomies compared to preoperative thoracic epidurals does not reduce hospital length of stay or postoperative narcotic usage. Patients who received cryoablation may still require an epidural to assist in their pain management regiment in the immediate postoperative period. Further studies are needed to elucidate the benefits of cryoablation in the pain management paradigms for patients who undergo thoracotomies.

## Data Availability

The data underlying this article will be shared on reasonable request to the corresponding author. **Kian Pourak:** Conceptualization; Methodology; Formal analysis; Investigation; Data Curation; Writing—Original Draft; Writing—Reviewing and Editing; Project administration. **Rachel Kubiak:** Investigation; Data Curation; Writing—Original Draft; Writing—Reviewing and Editing. **Kumaran Arivoli:** Investigation; Data Curation; Writing—Original Draft; Writing—Reviewing and Editing. **Kiran Lagisetty:** Validation; Writing—Reviewing and Editing; Supervision. **William Lynch:** Validation; Writing—Reviewing and Editing; Supervision. **Jules Lin:** Validation; Writing—Reviewing and Editing; Supervision. **Andrew Chang:** Validation; Writing—Reviewing and Editing; Supervision. **Rishindra M. Reddy:** Conceptualization; Methodology; Investigation; Writing—Review and Editing; Supervision; Project administration. Interactive CardioVascular and Thoracic Surgery thanks Larry R. Kaiser, Amit Bhargava and the other anonymous reviewers for their contribution to the peer review process of this article.

## Notes

Presented at the General Thoracic Surgical Club Annual Meeting, Duck Key, FL, USA, 9–12 March 2023.

## ABBREVIATIONS

DLT: Double-lung transplant
LR: Lung resection
MME: Morphine milligram equivalents
TTHHR: Transthoracic hiatal hernia repair
